# Integration of Machine Vision Technology in the Treatment of Pediatric Spinal Deformities

**DOI:** 10.7759/cureus.86790

**Published:** 2025-06-26

**Authors:** Zachary R Visco, Nathaniel C Adams, Alexandra N Johnson, Joseph D Stone, James O Sanders, Stuart L Mitchell

**Affiliations:** 1 Department of Orthopaedics, School of Medicine, The University of North Carolina at Chapel Hill, Chapel Hill, USA; 2 Department of Orthopaedics, Ballad Health, Johnson City, USA

**Keywords:** 7d surgical system, intraoperative navigation, new technology in spine surgery, pediatric orthopaedics, posterior spinal fusion

## Abstract

Aim: The study aimed to describe the use of the 7D FLASH Surgical System (Orthofix, Lewisville, USA), an intraoperative navigation tool, for complex pediatric spinal surgery. The authors present their techniques and recommendations for successfully implementing and utilizing this system.

Methods: We retrospectively identified all pediatric orthopaedic spinal surgeries utilizing the 7D FLASH System between July 2022 and January 2024. Pediatric patients with various spinal pathologies who underwent surgery by our institution's pediatric orthopaedic surgeons were included. Data collected encompassed surgical indications, instrumented levels, and 7D imaging registration information. The 7D data included the number of FLASH image registrations per case, the number of “FLASH Fixes” performed per case, and the average time required for each registration. These data were summarized using descriptive statistics. We also describe our intraoperative workflow and technical pearls in detail.

Results: Around 59 patients underwent posterior spinal fusion, and one patient had a cervical intracanal osteochondroma resection. A total of 644 levels were fused, with an average construct length of 10.91 ± 3.55 levels. The average device registration time was 59.36 ± 20.38 seconds. For the patients undergoing fusion, 1.53 ± 0.83 levels were instrumented per registration event. Around 22 patients required at least one “FLASH Fix” (n = 29 total Fixes), taking an average of 16.54 seconds per “FLASH Fix.”

Conclusions: Surgical navigation has become increasingly prevalent in recent years, although it typically necessitates the use of one or more intraoperative computed tomography (CT) scans. The quick device registration times and the capacity to instrument multiple levels per registration event imply that machine vision navigation technology may enhance intraoperative workflow efficiency compared to traditional intraoperative CT-based navigation, potentially reducing anesthesia time and radiation exposure.

Level of evidence: The study is classified as level IV evidence.

## Introduction

Pediatric spinal deformity surgery has seen numerous advancements since its inception [1]. These advancements include improvements in surgical instrumentation, perioperative patient optimization, and postoperative treatment protocols [1,2]. Notably, advancements in intraoperative management have had arguably the most significant effects on the safety and efficacy of pediatric spinal deformity surgery. For instance, intraoperative neuromonitoring has become standard practice in pediatric spinal surgery, providing essential information to surgeons that allows near real-time safety monitoring [3]. Stereotactic surgery is one technology that has gained prominence in recent years due to its ability to assist with the safe placement of spinal instrumentation. Horsley and Clarke produced one of the first stereotactic systems in an attempt to better understand cranial anatomy in the early 1900s [4]. This technology has been significantly improved and adapted over the past 100 years and is now utilized for the precise placement of spinal surgical tools and instrumentation with the goal of reducing or eliminating aberrant screw placement [2,3,5]. This is particularly important in patients with adolescent idiopathic scoliosis and more complex spinal etiologies, including congenital scoliosis, neuromuscular scoliosis, and intracanal pathologies. Furthermore, it is essential to critically evaluate emerging technologies and their impact on operative workflow in pediatric spinal deformity.

Modern surgical guidance systems rely on preoperative or intraoperative imaging, combined with intraoperative bony landmarks to identify safe trajectories for screw placement in real-time [2]. Numerous intraoperative navigation systems are commercially available, with many traditional systems requiring the use of an intraoperative computed tomography (CT) scan, such as Medtronic’s O-arm and StealthStation® System (Medtronic, Minneapolis, USA) or Stryker’s Airo TruCT and Ortho Q Guidance System (Stryker, Kalamazoo, USA) [6]. These systems demonstrate efficacy in terms of safe pedicle screw placement compared to traditional C-arm fluoroscopy; however, they often experience lower overall intraoperative efficiency metrics, such as increased operative times, which can lead to greater blood loss and infection rates [7-9]. For example, traditional systems require re-spinning a CT sequence to re-register patient anatomy if the stereotactic markers become disrupted, impeding operative flow [10]. Concerns over these efficiency barriers have limited more universal adoption, especially among senior surgeons with extensive experience using freehand or fluoroscopic-guided screw placement. Additionally, because traditional navigation systems rely on pre- and intraoperative imaging, there are concerns for patients and care teams undergoing repeated exposure to ionizing radiation [10]. Recently, a newer generation of image-guided navigation systems has been developed that relies on machine vision, a technology that utilizes visible or infrared light to register the patient’s anatomy with an imaging study. Navigation guidance is particularly beneficial in the pediatric spine patient population given the relatively high rate of congenital abnormalities and dysplastic vertebrae, allowing for more accurate screw placement than traditional non-navigated screws [11,12]. Machine vision navigation has gained popularity due to its relative ease of use and its ability to utilize a preoperative CT scan for intraoperative patient registration, leading to improved operative efficiency and lower radiation exposure for the surgical team (compared to fluoroscopic guidance) [5,10,13]. 

Machine vision navigation systems utilize a stereotactic light camera system to identify bony anatomy using machine learning technology, which is then registered with a preoperative CT scan [5]. A separate infrared camera system, similar to traditional navigation systems, then tracks surgical instruments in space, producing a real-time view of instrument trajectory overlaid on the preoperative CT scan [14]. The 7D FLASH Surgical System (Orthofix, Lewisville, USA) is one of these novel machine vision navigation systems developed for use in adult and pediatric spinal surgery [15]. The purpose of this study was to evaluate the use of the 7D FLASH System for the treatment of complex pediatric spinal pathologies (predominantly adolescent idiopathic, neuromuscular, and congenital scoliosis repairs) and to offer techniques and recommendations for best practices in the implementation and utilization of machine navigation systems at other institutions.

## Materials and methods

Data collection

A retrospective review was conducted for all pediatric spinal surgical cases performed at our tertiary referral center, UNC Hospitals, Chapel Hill, USA, between July 2022 and January 2024, using the 7D FLASH Surgical System by our institution’s fellowship-trained pediatric orthopaedic surgeons. The Institutional Review Board's approval was obtained. Data collected for each surgical case included surgical indication, surgical details, and 7D imaging registration information. The 7D data comprised the number of FLASH image registrations performed per case, the number of “FLASH Fixes” (described below) executed per case, and the average time required for each registration. These data were summarized using descriptive statistics due to the heterogeneous nature of the patients' pathologies in this cohort.

7D FLASH Surgical System workflow

The 7D FLASH System relies on a CT scan for reference imaging, typically obtained preoperatively. This system employs a low-dose, high-resolution CT scan with a 1-2 mm slice thickness that includes at least one vertebra above and below the specific levels of interest [16]. The preoperative CT scan is then subjected to a 3D reconstruction on the 7D System for intraoperative use. This scan is loaded onto the 7D FLASH device workstation before the surgical case begins. If preferred, an intraoperative CT scan can be obtained and loaded onto the system if the surgeon opts against a preoperative CT scan. The 7D System also features FLASH Navigation MRVision, which utilizes a preoperative magnetic resonance image (MRI) for intraoperative navigation.

**Figure 1:**
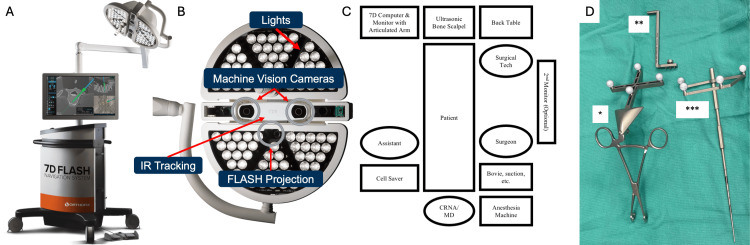
(A) The 7D FLASH Surgical System's non-sterile device workstation. (B) The 7D FLASH Surgical System's overhead surgical light and machine vision navigation system with built-in stereotactic cameras, IR cameras, and the FLASH projection system. (C) Standard intraoperative room setup with the 7D FLASH device workstation positioned at the foot of the bed. (D) The 7D FLASH Surgical System's instruments: flex array (*), which can be clamped to bony landmarks (e.g., spinous processes) with attached IR probes; flex rod connector (**), which can be connected to a tulip head screw and then attached to a flex array for tracking; and navigated ball-tip pointer (***). IR: Infrared; CRNA/MD: Certified registered nurse anesthetist/medical doctor Figures (A, B) have been adapted from Orthofix's 7D FLASH System Brochure with permission [13]. Figures (C, D) were created by the authors.

The surgeon performs routine positioning, prepping, and draping in a standard manner at the start of each surgical case. Device representatives prepare the 7D FLASH device workstation for utilization while exposure is completed. The workstation is a non-sterile mobile computer and monitor combined with a surgical light and camera system on an articulated arm (Figures 1A-1B). The system includes a foot pedal and can be operated entirely by the surgeon, but we prefer to have the device representative run the system for efficiency and enhanced ergonomics. The senior author prefers placing the 7D System at the foot of the bed, although this is not strictly necessary (Figure 1C). The 7D display can also be linked to additional monitors in the room, allowing assistants and surgical trainees to easily view the screen. While the spine is exposed by the surgical team, the surgical technologist works with the device representative to calibrate instruments. Each surgical instrument has a reference array of reflective fiducial markers identified using stereoscopic infrared cameras for spatial tracking intraoperatively (Figure 1D). The entire spine can be exposed first as per routine, prior to navigation and instrumentation. Surgical dissection resembles traditional surgical approaches and does not necessitate radical surgical exposure [5]. The facet capsules can be left in place if needed by the surgeon, and there is no requirement for a wide lateral exposure [5]. Additionally, facetectomies can be performed prior to navigating, allowing more than sufficient bone for accurate image acquisition and navigation.

**Figure 2:**
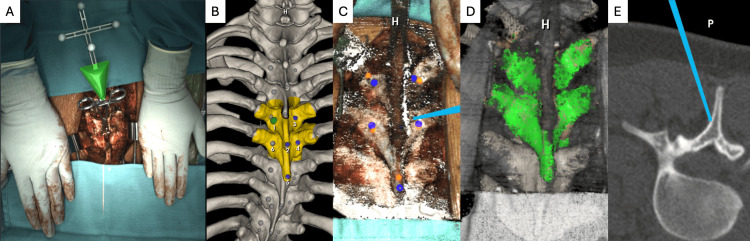
(A) Intraoperative view of an exposed thoracic spine as seen by the machine vision system, with the flex array highlighted in green, indicating appropriate device registration. Surgical towels placed cranially and caudally cover other exposed spinal segments, while the surgeon’s hands cover reflective surgical retractors to optimize device registration. (B) The 3D reconstruction of a patient’s preoperative CT scan with T5 and T6 highlighted for device registration using machine vision navigation. Numbered dots are used to mark specific bony landmarks, which are then selected by the surgeon using a navigated probe. (C) A navigated probe (blue line) is used to select landmarks on the patient (orange dots), which are then correlated to the landmarks identified on the CT scan (blue dots). (D) Device registration of the intraoperative spinal topography overlaid onto the preoperative CT scan. (E) Projection of the navigated probe marking a thoracic lamina onto the intraoperative CT scan. CT: Computed tomography

A flex array is clamped to a bony landmark (e.g., spinous process) or previous instrumentation to initiate the navigation process. Once the flex array is positioned, the system captures a “FLASH” using the overhead surgical light with stereotactic visible light cameras to create a 3D topographical map of the spine (Figure 2A). We generally calibrate the system by marking the spinous process and bilateral lamina of each vertebra (Figures 2B-2D), but the surgeon may select any visible bony landmark. The supraspinous ligament may cause a registration error if left in place, so an electrocautery device is employed to create a pathway through the ligament and apophysis before registration, ensuring the navigated probe contacts the bony surface directly. The 7D System then utilizes machine learning to match the surface topography on the FLASH image data with the preoperative CT scan (Figure 2D). The device registration is checked by selecting bony landmarks with a navigated probe and comparing them to the projection of the probe on the preoperative CT scan (Figure 2E). When the device is accurately registered, the projection of the navigated probe is positioned directly on the surface of the bone. An incorrect registration will display the tip of the navigated probe above or below the surface of the bone or translated medially or laterally relative to its true location.

**Figure 3:**
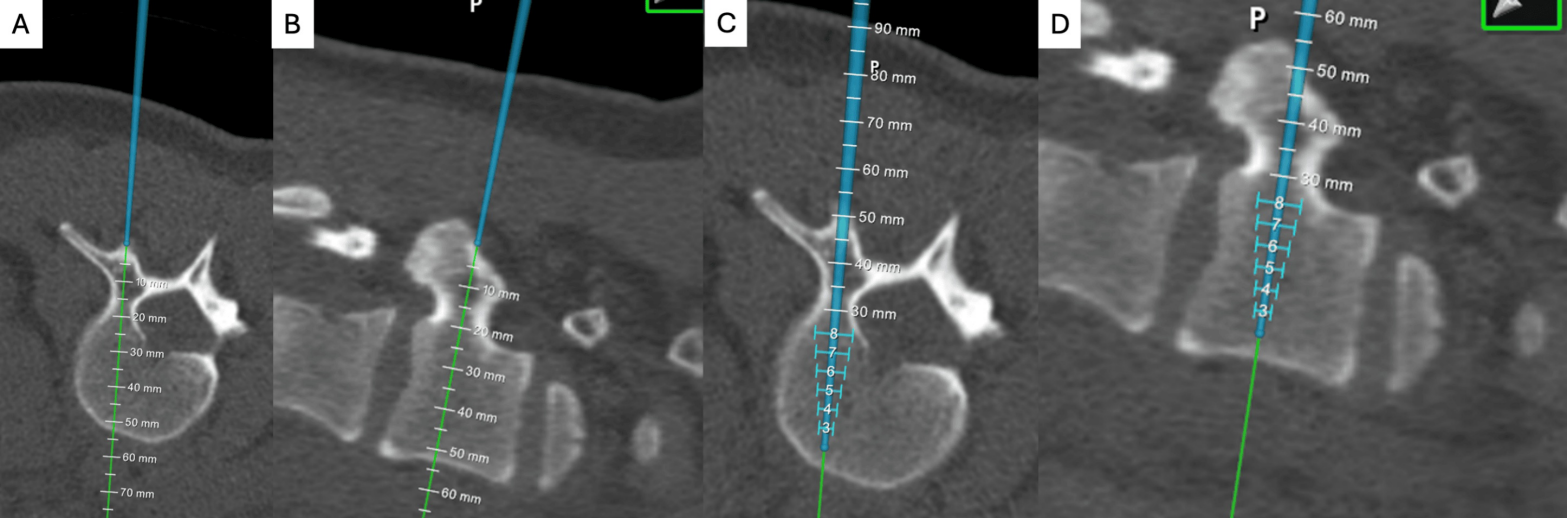
(A) A navigated probe is used to map the optimal trajectory of a pedicle screw on an axial slice of the preoperative CT scan after identifying anatomic landmarks with a traditional freehand technique. (B) A navigated probe is used to map the optimal trajectory on a sagittal slice of the preoperative CT scan. (C) A navigated awl with projected screw lengths overlaid onto an axial slice of the preoperative CT scan. (D) A navigated awl with projected screw lengths overlaid onto a sagittal slice of the preoperative CT scan. CT: Computed tomography

Once the system is calibrated, the surgeon can begin preparing pedicles for instrumentation. The 7D System permits navigated instrumentation using various instruments, including navigated awls, drills, and probes, among others. The senior author prefers to perform a navigation-assisted freehand technique. In this technique, the surgeon identifies anatomic landmarks using a traditional free-hand method and then employs the navigated probe to optimize the location and orientation to find the largest possible tract (Figures 3A-3B). The pedicle is then cannulated using either a navigated awl or a flexible 2.0 mm reamer on pulse-oscillation (Figures 3C-3D) [16]. The 7D System displays screw diameter and lengths on the screen and can be utilized to estimate the screw size. Pediatric pedicles can dilate up to 73%, yet the length remains constant following cannulation [17]. The screw length measurement is generally accurate, but the screw diameter is often underestimated. We typically use 6.5 or 7.0 mm screws in the lumbar and distal thoracic spine and 5.0 or 5.5 mm screws in the remaining thoracic spine [18]. Pedicle tracts can be tapped and screws placed with or without navigation assistance, but we prefer to operate without navigation assistance for efficiency and ease. If registration becomes inaccurate, a “FLASH Fix” can be executed in seconds to quickly recalibrate the system if needed. This involves capturing a new FLASH image of the spine without the necessity to mark bony landmarks again. In the unlikely event that a “FLASH Fix” fails to restore accuracy, the system can be re-registered using the sequence of steps outlined above. Fluoroscopy films are obtained at the end of the case to confirm safe screw position and trajectory.

## Results

In total, 60 pediatric spinal surgeries were performed using the 7D FLASH System at our institution during the data collection period. Around 59 (98%, n = 60) patients underwent posterior spinal fusion (PSF) (patient conditions listed in Table 1), while one patient had a cervical intracanal osteochondroma resection. A total of 644 levels were fused (n = 59), with an average construct length of 10.91 ± 3.55 levels. Pelvic fixation was carried out in nine cases (15%, n = 59). Two patients with early-onset scoliosis (EOS) and one patient with infantile scoliosis underwent short-segment fusions at the curve apex as part of the Shilla guided-growth procedure. The average device registration time from taking the "FLASH" to accepting device registration was 59.36 ± 20.38 seconds. For the PSF cases, 1.53 ± 0.83 levels were instrumented per registration event. Around 22 patients required at least one “FLASH Fix” (n = 29 total fixes), taking an average of 16.54 seconds per “FLASH Fix.” 

**Table 1:** Condition of the patients who underwent posterior spinal fusion (n = 59). Data has been represented as: n = number of patients with given condition; (%) = proportion of patients with that condition that make up the posterior spinal fusion group.

Condition	No. of Patients, n (%)
Adolescent idiopathic scoliosis (AIS)	24 (41%)
Neuromuscular/syndromic scoliosis	16 (27%)
Early-onset scoliosis (EOS)	7 (12%)
Congenital scoliosis	4 (7%)
Juvenile/infantile scoliosis	4 (7%)
Scheuermann’s kyphosis	2 (3%)
Isthmic spondylolisthesis	1 (1.5%)
Spina bifida	1 (1.5%)

## Discussion

Machine vision navigation is particularly well-suited for treating complex pediatric spinal deformities [2,19]. The performance and intraoperative usability of the 7D FLASH System were evaluated in this case series. It offers several significant advantages over both 2D fluoroscopy and traditional image-guided navigation systems. This case series has enabled our institution’s surgeons to become familiar with the 7D System and identify key surgical pearls and limitations for its successful implementation at other institutions. 

The 7D System is equipped with an overhead light, which also includes embedded stereotactic cameras, each angled at 45 degrees converging on the surgical field, along with infrared instrument trackers. Our surgeons noted that typical operating room (OR) lights negatively affect 7D registration due to overexposure of the surgical field; hence, the 7D surgical light was generally used without the standard overhead OR lights. Additionally, reflections from the light on metallic objects in the field of view can generate interference, so these should be covered with sterile towels before registration (Figure 2A). Optimization of the “FLASH” can be achieved by aligning the spine rotation with the light/camera position. This is particularly helpful during block registration, as it allows for multiple levels to be calibrated simultaneously. To register additional surgical levels with the 7D System, one simply moves the calibration array and repeats the device registration process. This system also benefits from its ability to perform a “FLASH Fix” if the reference array is disturbed, unlike traditional navigation systems, which require a repeat CT scan if device calibration is disrupted. Prior studies have indicated that traditional navigation and robotic-assisted pedicle screw placement allow for safe and accurate placement of pedicle screws, though they often require longer operative times [20,21]. Our study evaluating machine vision technology demonstrated short registration times, infrequent need for “FLASH Fixes,” and the capacity to instrument multiple levels using a single registration. This allowed surgeons to efficiently instrument the spine without the need to repeat lengthy intraoperative CT scans, potentially decreasing intraoperative time compared to traditional navigation systems. Future research is needed to evaluate machine vision surgical times against traditional navigation systems and free-hand techniques. 

Traditional navigation suffers from the “drift phenomenon,” during which the system struggles to correlate intraoperative information with the CT scan as the spine shifts into a new alignment during instrumentation and correction [22]. This results in discrepancies between the projected screw trajectory and the actual screw trajectory, increasing the risk of instrumentation misplacement and pedicle breaches [18]. Addressing this issue intraoperatively often requires a repeat CT scan, which can negatively affect intraoperative workflow and efficiency. The 7D System allows users to perform a “FLASH Fix” if the reference array is disturbed or if a “drift” is noted during the procedure. This system is typically registered with one or two vertebrae at a time against the preoperative CT scan, with the possibility of including up to five vertebrae. Since the orientation of single levels does not change significantly, the 7D System is resistant to the so-called “drift phenomenon” as corrections are made, even in a flexible pediatric spine. Consequently, the entire navigation process can be executed using a single CT scan, regardless of the extent of deformity corrected, the length of the planned surgical construct, or the completion of all facetectomies prior to instrumentation. 

Optical navigation systems have wide-ranging utility, as demonstrated by our case series. In our pediatric orthopaedic setting, the 7D System has been most frequently used for treating AIS, neuromuscular scoliosis, and sagittal deformities. The rapid device registration facilitates safe and accurate placement of pedicle screws without the use of traditional fluoroscopy at every level. However, after completing the construct, our surgeons use either C-arm fluoroscopy or flat-plate X-ray to confirm alignment and hardware position. These systems allow users to confidently place screws into dysplastic pedicles and safely position pelvic instrumentation if necessary. Furthermore, they assist with highly technical surgical dissection around the spinal cord, as utilized by one of our surgeons in the removal of an intracanal cervical osteochondroma. In patients with previous instrumentation, the system can be registered off prior pedicle screws or rods, particularly if the spinous processes have been removed. These systems are adjustable and versatile, enabling them to assist in treating various complex pediatric surgical pathologies.

Despite its numerous benefits, optical navigation systems cannot replace a highly trained surgeon. They enhance a surgeon’s understanding and interpretation of 3D bony anatomy, but still require the surgeon to apply clinical judgment to confirm screw position and rule out pedicle breaches. In patients with complex transitional anatomy or bony overgrowth during revision surgery, machine vision can struggle to interpret bony landmarks. As a result, the system can still experience the “drift phenomenon,” potentially leading to inadvertent screw placement. In these cases, our surgeons typically use standard fluoroscopy for instrumentation and screw position confirmation rather than relying solely on the machine vision system. It is also crucial that the system is registered to the correct level, since certain levels, especially in the thoracic spine, can exhibit similar surface topography. Although the system will attempt to register, it may appear less accurate; thus, it is the surgeon’s responsibility to confirm the appropriate levels prior to registration. Optical navigation systems also were not originally designed for minimally invasive percutaneous procedures, but ongoing advances in technology have shown promising results for such techniques [23]. 

This study is not without limitations. The goal of this paper was to share the authors' techniques and recommendations for successfully implementing and utilizing an optical navigation system in pediatric spine deformity surgery. As this paper focused exclusively on the technique, intraoperative neuromonitoring and patient outcome data were not collected. This limits the ability to analyze outcomes between this cohort and those who received instrumentation through more traditional navigation or fluoroscopic guidance. Furthermore, this study was conducted at a tertiary referral academic institution, which may limit generalizability to some extent. There are institutional costs and learning curves associated with adopting any new surgical technology. Surgical navigation systems often entail a significant upfront cost, which is theoretically paid over time through improvements in intraoperative efficiency [2]. The time required to become proficient with optical navigation systems may be prohibitive for surgeons who do not have dedicated time to learn new technologies. However, it is possible to achieve proficiency in pediatric scoliosis repairs in as few as nine cases, which can be a reasonable timeframe for many surgeons [24].

## Conclusions

Surgical navigation has gained increased prominence in recent years as the technology has advanced and more systems have become available. Our experience with the 7D FLASH Surgical System indicates that it can be easily integrated into pediatric spinal deformity workflows, though its clinical benefits still require further validation. This study did not evaluate patient outcomes or compare screw placement accuracy against traditional navigation or freehand methods. Future prospective work is necessary to quantitatively assess the safety, implementation cost, and patient-reported outcomes associated with the use of machine vision technology before it can be formally recommended for broad inclusion in all pediatric spine surgeries.
